# SNAP-tagged Chikungunya Virus Replicons Improve Visualisation of Non-Structural Protein 3 by Fluorescence Microscopy

**DOI:** 10.1038/s41598-017-05820-0

**Published:** 2017-07-18

**Authors:** Roland Remenyi, Grace C. Roberts, Carsten Zothner, Andres Merits, Mark Harris

**Affiliations:** 1 School of Molecular and Cellular Biology, Faculty of Biological Sciences, and Astbury Centre for Structural Molecular Biology, University of Leeds, Leeds, West Yorkshire LS2 9JT United Kingdom; 2 Institute of Technology, University of Tartu, Tartu, Estonia

## Abstract

Chikungunya virus (CHIKV), a mosquito-borne alphavirus, causes febrile disease, muscle and joint pain, which can become chronic in some individuals. The non-structural protein 3 (nsP3) plays essential roles during infection, but a complete understanding of its function is lacking. Here we used a microscopy-based approach to image CHIKV nsP3 inside human cells. The SNAP system consists of a self-labelling enzyme tag, which catalyses the covalent linking of exogenously supplemented synthetic ligands. Genetic insertion of this tag resulted in viable replicons and specific labelling while preserving the effect of nsP3 on stress granule responses and co-localisation with GTPase Activating Protein (SH3 domain) Binding Proteins (G3BPs). With sub-diffraction, three-dimensional, optical imaging, we visualised nsP3-positive structures with variable density and morphology, including high-density rod-like structures, large spherical granules, and small, low-density structures. Next, we confirmed the utility of the SNAP-tag for studying protein turnover by pulse-chase labelling. We also revealed an association of nsP3 with cellular lipid droplets and examined the spatial relationships between nsP3 and the non-structural protein 1 (nsP1). Together, our study provides a sensitive, specific, and versatile system for fundamental research into the individual functions of a viral non-structural protein during infection with a medically important arthropod-borne virus (arbovirus).

## Introduction

Chikungunya virus (CHIKV), an arthropod-borne virus (arbovirus) of the *Alphavirus* genus, causes rapid-onset fever accompanied by fatigue, joint/muscle pain, headaches, and rashes in infected individuals^1^. Moreover, joint pain can persist for years^2^. This disease burden presents a challenge to public health systems, especially in low- and middle-income countries. Moreover, global travel may facilitate the further spread of CHIKV, while climate change may expand the habitat of *Aedes aegypti* and *Aedes albopictus* mosquitoes, which transmit the virus to humans^3^. Numerous vaccine candidates are in development, but the explosive nature of CHIKV epidemics may complicate efficacy testing in humans^4^. Various reports link anti-CHIKV activity to drug candidates (for a review, see ref. 5), but the mechanism-of-action and *in vivo* efficacy of these inhibitors needs further evaluation. Thus, there remains an unmet need to prevent and treat CHIKV infections effectively.

The 11.8-kilobase, single-stranded, positive-sense-RNA genome of CHIKV encodes four non-structural proteins (nsP1, nsP2, nsP3, nsP4) and three main structural proteins (capsid and envelope glycoproteins E1/E2). The genome also encodes three additional structural proteins E3, 6 K, and transframe (TF)^6–8^, but these proteins are not always present in mature virions. The study of related Sindbis virus (SINV), Semliki Forest virus (SFV), Eastern equine encephalitis virus (EEEV) and Venezuelan equine encephalitis virus (VEEV) has given invaluable insight into alphavirus infection, but much remains to be learned about the specifics of CHIKV replication and the host-cell response^1^. Although recent work on nsP3 establishes an essential enzymatic function as an ADP-ribosylhydrolase^9, 10^, a complete picture of distinct roles of nsP3 during the CHIKV life cycle has yet to emerge. The suggested roles of nsP3 include viral RNA synthesis^11–15^, targeting of replication complexes to endolysosomal vesicles^16^, inclusion in replication complexes at the plasma membrane^17^, suppression of host antiviral pathways (reviewed in ref. 18), activation of cellular signalling pathways^19^, and reversion of protein ADP-ribosylation (through hydrolysis of mono [ADP-ribose])^9, 10, 20–22^. Post-translational modification of nsP3 by phosphorylation plays a role in SINV negative-strand synthesis but has a minor effect on SFV and VEEV replication^12, 23–26^. How the protein can carry out this multitude of roles inside the host cell remains unknown. Because various studies uncovered differences in the function of nsP3 between related alphaviruses^19, 26, 27^, there is a need to characterise CHIKV-specific roles for nsP3.

NsP3 consists of three domains: a highly conserved N-terminal macrodomain (containing the enzymatic ADP-ribosylhydrolase activity^9, 10^), a middle region with zinc-binding sites^28^, and a hypervariable C-terminus. This C-terminal domain contains multiple phosphorylation sites, is intrinsically disordered, and lacks a defined secondary structure. Moreover, specific sequence motifs within the C-terminal domain are required to recruit cellular host factors such as Amphiphysin-1 and -2 (Amph1 and 2) and the GTPase Activating Protein (SH3 Domain) Binding Protein (G3BP)^27, 29–39^. This domain can also accept insertion of foreign proteins such as luciferase, green fluorescent protein (GFP), and mCherry^26, 30, 31, 40–43^. These fluorescent CHIKV derivatives alongside traditional immunostaining approaches have allowed various groups to visualise the subcellular distribution of nsP3^19, 27, 31, 32, 37, 40^. Compared to fluorescent proteins, self-labelling tags offer added flexibility through compatibility with fluorescent probes with superior physicochemical properties^44^. The self-labelling SNAP-tag uses the enzyme O^6^-alkylguanine transferase fused to the protein of interest^44, 45^. The addition of benzylguanine-tethered fluorophores covalently joins the fluorophore to the SNAP-tagged fusion protein^44^. The SNAP-tag technology moderately reduces the overall label size (by about 1/3) and importantly facilitates measurements of protein turnover in cells and animals^46, 47^. Our laboratory has previously used this SNAP technology to tag the non-structural protein 5 A (NS5A) of hepatitis C virus and to image multiple replicons in the same host cell by fluorescence microscopy^48^.

In this study, we used the SNAP system to tag the nsP3 protein of CHIKV in the context of a replicon. We confirmed that a replicon encoding SNAP-tagged nsP3 replicated in human hepatic cells and that SNAP-nsP3 formed granular clusters as well as rod-like structures. Furthermore, time-dependent, pulse-chase staining of the SNAP-tag showed that CHIKV nsP3 accumulated in intracellular granules rather than rod-like structures. We combined the improved labelling system with sub-diffraction, three-dimensional (3-D), optical imaging. The resulting images provided evidence for an association of nsP3 with cellular lipid droplets and revealed high-resolution spatial relationships between nsP3 and nsP1. Thus, this study expands the portfolio of CHIKV reagents and provides an improved tool for fundamental research studies to identify and track a multi-functional protein in the complex cellular environment. In turn, efforts to develop anti-CHIKV treatments will benefit from an improved understanding of CHIKV biology, including a better grasp of the various roles of nsP3 that antiviral strategies could target in the future.

## Results

### Tagging of nsP3 with the SNAP-sequence results in viable CHIKV replicons for specific labelling with fluorescent probes

**Figure 1:**
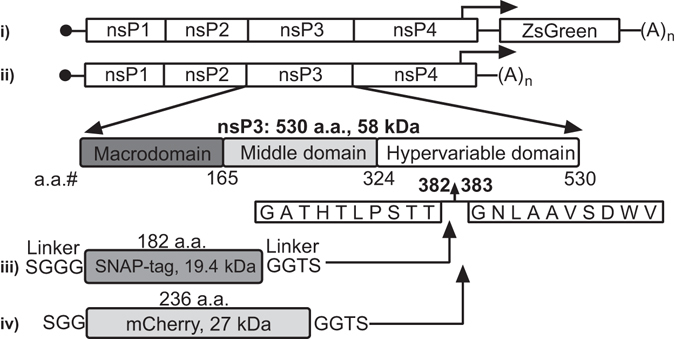
Inserting the SNAP polypeptide into the hypervariable domain of CHIKV nsP3. Schematic representation of replicons with a ZsGreen reporter gene (**i**) and without (**ii**). We inserted the SNAP-tag sequence between the codons that encode amino acids 382 and 383 of nsP3 (in the hypervariable domain, **iii**). Flexible linker-peptides that flank the SNAP and an mCherry (**iv**) insertion are shown as well, alongside the mass of the protein insertion (in kilodaltons, kDa) and the number of amino acid residues (a.a.).

To visualise nsP3 by advanced light microscopy techniques, we cloned the SNAP-tag sequence between the codons for amino acid residues 382 and 383 with a linker peptide flanking the in-frame insertion (Fig. 1, iii). CHIKV replicon systems either contained the ZsGreen gene under the control of the CHIKV subgenomic (sg) promoter (Fig. 1, i), or lacked the region that encodes the structural proteins (Fig. 1, ii). Parental replicons are referred to hereafter as CHIKV^repl^ and CHIKV^repl sg-ZsGreen^. Furthermore, the nomenclature CHIKV^repl SNAP-P3^, CHIKV^repl mCherry-P3^, CHIKV^repl SNAP-P3 sg-ZsGreen^, and CHIKV^repl mCherry-P3 sg-ZsGreen^ was chosen for replicons containing SNAP-nsP3 or mCherry-nsP3 (Fig. 1, iv). Note that the non-infectious replicon system permitted the study of nsP3 function and handling of live cells outside of a biocontainment facility.

**Figure 2:**
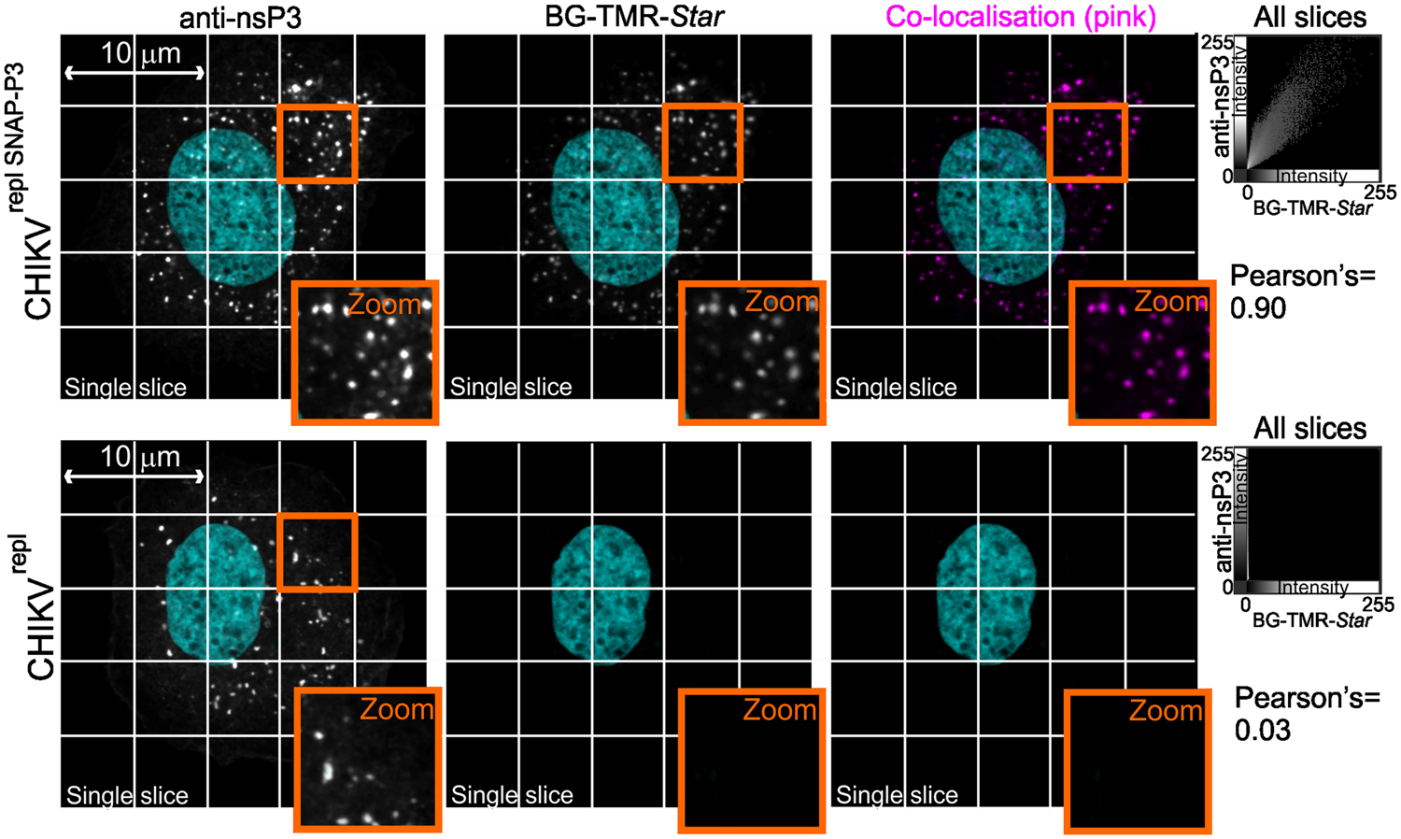
Inserting the SNAP polypeptide into the CHIKV replicon allows for specific labelling of nsP3 with synthetic probes. Confocal microscopy of fixed samples labelled for nsP3 and the SNAP-tag. HuH-7 cells were transfected with SNAP-tagged CHIKV^repl SNAP-P3^ RNA or untagged CHIKV^repl^ RNA. BG-TMR-*Star* substrate stained SNAP-nsP3, rabbit antiserum detected nsP3, and Z-stacks were acquired by confocal microscopy. Individual images show single, central slices extracted from the Z-stack. Scatterplots show fluorescence intensity of all voxels from nsP3-specific and BG-TMR-*Star*-specific imaging channels (for entire Z-stack). Note that for CHIKV^repl^, the data points line up along the y-axis due to low fluorescence intensity in the BG-TMR-*Star* channel. Pearson’s = Pearson’s correlation coefficient. Imaris software was used to extract single slices from Z-stacks, create the pseudo-coloured co-localisation channel (pink) and to plot fluorescence intensities on 2-D scatterplots. Data was acquired with a Zeiss LSM 700 confocal system equipped with a Plan-Apochromat 63× oil objective (1.4 numerical aperture).

To confirm that SNAP-specific substrates could label the SNAP-nsP3 fusion protein, we transfected HuH-7 cells with CHIKV^repl SNAP-P3^ RNA and stained fixed cells with the fluorescent TMR-*Star*-conjugated O6-benzylguanine probe (BG-TMR-*Star*). The choice of the human hepatoma cell line HuH-7 for our studies is discussed in more detail in the supplementary information (Supplementary Text S1). Immunostaining with antiserum raised against CHIKV nsP3 revealed an overlap between the BG-TMR-*Star* signal and the staining pattern generated by the nsP3-specific antiserum (Fig. 2). This co-localisation was also supported by a high Pearson’s correlation coefficient (=0.9). In comparison, we only detected low-level background signal for BG-TMR-*Star* in cells transfected with wild-type CHIKV^repl^ RNA (Fig. 2). Thus, these immunostaining assays confirmed that SNAP-specific probes could label SNAP-nsP3 after transfection of CHIKV^repl SNAP-P3^ RNA.

**Figure 3:**
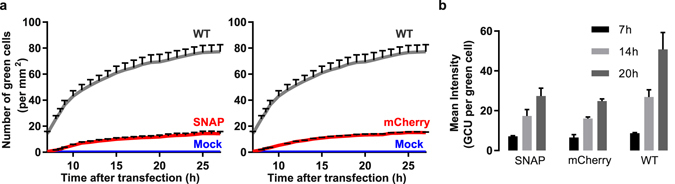
SNAP-tagged replicon retains replicative capacity. (**a**) Subgenomic RNA in HuH-7 cells transfected with CHIKV^SNAP-P3 sg-ZsGreen^ (SNAP), CHIKV^mCherry-P3 sg-ZsGreen^ (mCherry), and CHIKV^repl sg-ZsGreen^ (WT) replicons was quantified by measuring the level of ZsGreen protein. The level of green fluorescence was monitored hourly by live-cell imaging with an IncuCyte ZOOM instrument equipped with a 10× objective. Mean values are plotted alongside standard error of measurement (SEM) from three independent transfections, with nine images taken at indicated times for each replicate (7 h to 27 h after transfection). Data are presented as the number of green objects (cells) per mm^2^. Untransfected cells, which remained non-fluorescent, were included as a negative control (Mock). (**b**) Mean values of the fluorescence intensity in the green channel were calculated (in GCU, green calibrated units, per cell) with the instrument’s software. Averages at three time-points (7 h, 14 h, and 20 h after transfection) are plotted alongside standard error of measurement (SEM) from three independent transfections.

Next, we characterised the replicative fitness of SNAP-tagged replicons. To measure marker protein expression from subgenomic RNA, we quantified green fluorescence in live cells after transfection of CHIKV^repl SNAP-P3 sg-ZsGreen^ RNA. In this replicon, the ZsGreen gene is under the control of the CHIKV subgenomic promoter. Note that untransfected cells remained non-fluorescent in this experiment (for individual images, see Supplementary Fig. S1). Tagging nsP3 with mCherry or the SNAP-tag reduced the number of green cells (Fig. 3a and Supplementary Fig. S1) and the mean intensity of the green fluorescence per cell (Fig. 3b). However, a time-dependent increase in green cells and fluorescence intensity confirmed that the SNAP-tag did not have a deleterious effect on viral replication (Fig. 3). In conclusion, the SNAP-tagged and mCherry-tagged replicons had a similar replicative capacity, albeit at a lower level compared to an untagged replicon.

### Fusion of the SNAP-tag to nsP3 does not impair its known functions

**Figure 4:**
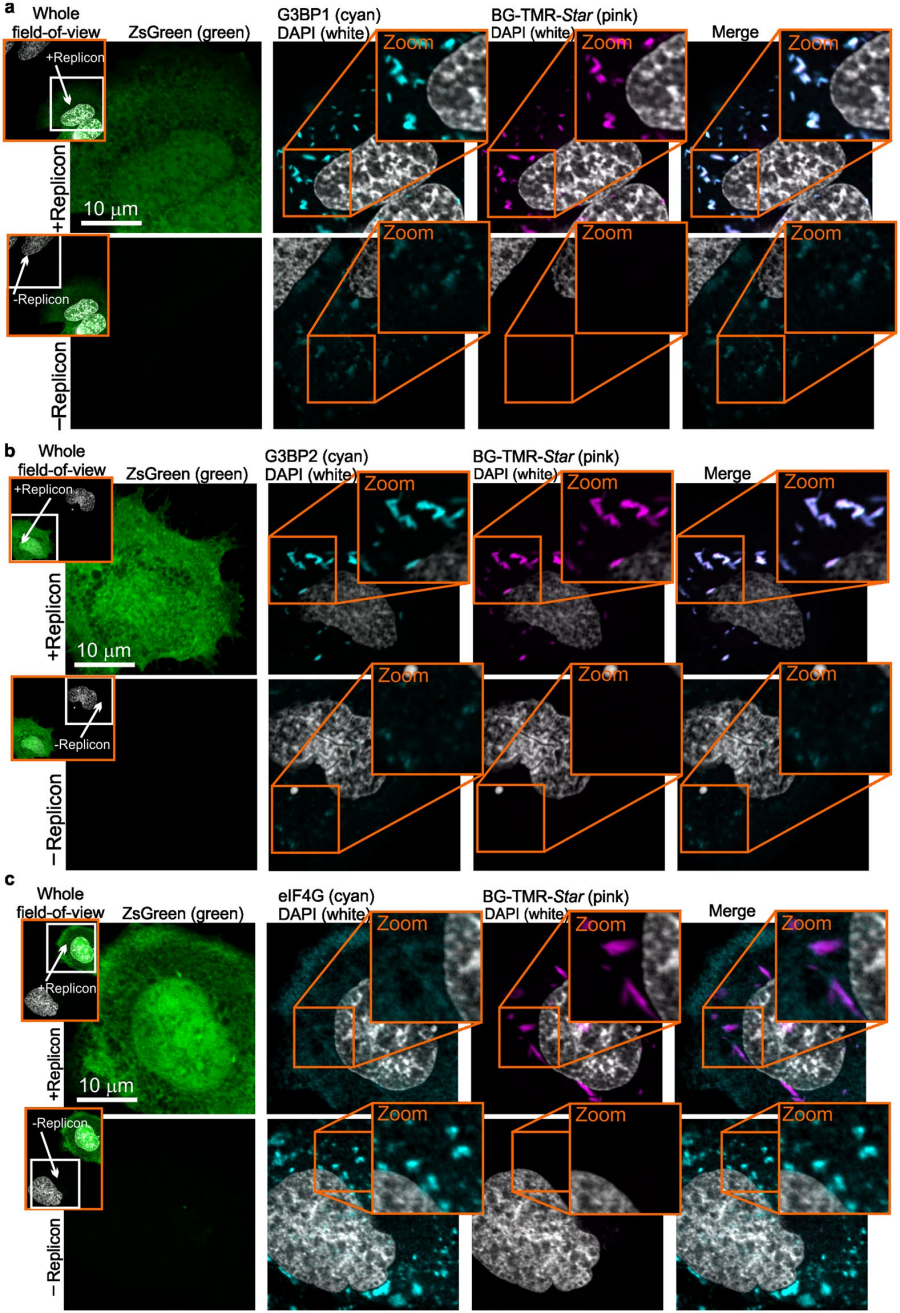
SNAP-nsP3 retains known function and interaction with G3BP1/2 cellular proteins. CHIKV^SNAP-nsP3 sg-ZsGreen^ RNA was transfected into HuH-7 cells by electroporation. After 11 h, stress granules were induced by sodium arsenite treatment (1 mM, 45 min), followed by fixation and staining for SNAP-nsP3 by BG-TMR-*Star* (pink). Nuclei were counterstained with DAPI (cyan). Data was acquired with a Zeiss LSM 880 confocal system with Airyscan, operated in the super-resolution mode with a Plan-Apochromat 63× oil objective (1.4 numerical aperture). (**a**) G3BP1 (cyan) was immunostained with mouse antibodies that bind to G3BP1. SNAP-nsP3 co-localised with G3BP1 in ZsGreen-positive cells. Inserts on the left show entire field-of-view from which individual regions were selected that either contained a ZsGreen-positive cell (+Replicon) or a ZsGreen-negative cell (−Replicon). Inserts on the right represent zoomed-in views of boxed regions. Rightmost images show overlays of the signals from the indicated fluorescent channels (G3BP1/2-specific signal pseudo-coloured in cyan, BG-TMR-*Star* in pink). (**b**) G3BP2 (cyan) was immunostained with rabbit antibodies that bind to G3BP1. SNAP-nsP3 co-localised with G3BP2 in ZsGreen-positive cells. (**c**) Stress granule marker eIF4G (cyan) was immunostained with antibodies that bind eIF4G. SNAP-nsP3 did not sequester eIF4G in ZsGreen-positive cells.

Next, we investigated whether SNAP-nsP3 can function in the same fashion as untagged nsP3. G3BP proteins form complexes with nsP3 proteins of Old World alphaviruses and the New World alphavirus EEEV^29–38, 49^. G3BPs are key components of stress granules^50^, which may function to support and in other cases inhibit viral infection (for a recent review, see ref. 51). During CHIKV infection, G3BPs play a pro-viral role^27, 37^. To test whether the interaction between G3BP proteins and nsP3 remained intact after insertion of the SNAP-tag, we transfected cells with CHIKV^repl SNAP-P3 sg-ZsGreen^ RNA, stained for SNAP-nsP3 with the BG-TMR-*Star* substrate, and labelled cells with antibodies that recognise the G3BP1 or G3BP2 isoforms. For these experiments, we used an emerging super-resolution optical microscopy approach. Briefly, Airyscan confocal super-resolution microscopy (Airyscan or Image Scanning Microscopy)^52, 53^ relies on photon-pixel-reassignment with an array detector to oversample the pattern produced by diffracted light and can improve image resolution in all spatial directions by 1.7-fold^54^. To induce stress granules via oxidative stress and compare intracellular stress granule responses between transfected and untransfected cells we treated live cells with sodium arsenite. We saw an induction of G3BP1/2-positive protein structures in ZsGreen-negative cells (Fig. 4a,b, “-Replicon” panels) and nsP3 co-localised with G3BP1 and G3BP2 in ZsGreen-positive cells (Fig. 4a,b, “+Replicon” panels). The presence of sodium arsenite, however, did not lead to the induction of additional G3BP1/2-positive granules in ZsGreen-positive cells (Fig. 4a,b, zoomed regions).

For CHIKV and SFV, nsP3 plays a role in the sequestration of G3BP proteins; this sequestration disassembles stress granules and interferes with antiviral stress-granule pathways^18, 31, 36^. Because the nsP3-G3BP1-G3BP2 protein structures induced by CHIKV lack additional canonical markers of stress granules, these structures are not viewed as genuine, *bona fide* stress granules^36, 37^. To test whether SNAP-nsP3 co-localised with a marker other than G3BP1/2, we probed cells with antibodies specific for eIF4G, a known component of genuine stress granules. Granular structures of eIF4G were readily detected in arsenite-treated, ZsGreen-negative cells (Fig. 4c, “-Replicon” panel). In contrast, eIF4G staining in arsenite-treated, ZsGreen-positive cells was diffuse and lacked the characteristic, high-density granules (Fig. 4c, “+Replicon” panel). Moreover, eIF4G did not accumulate in nsP3-positive structures. Collectively, our data suggested that fusion of the SNAP-tag to nsP3 did not affect the ability of nsP3 to interact with known binding partners or to carry out a previously described function, namely the interference with cellular stress-granule responses by sequestration of G3BPs.

### Cytoplasmic SNAP-nsP3 forms granular and rod-like structures while accumulating in granular structures over time

**Figure 5:**
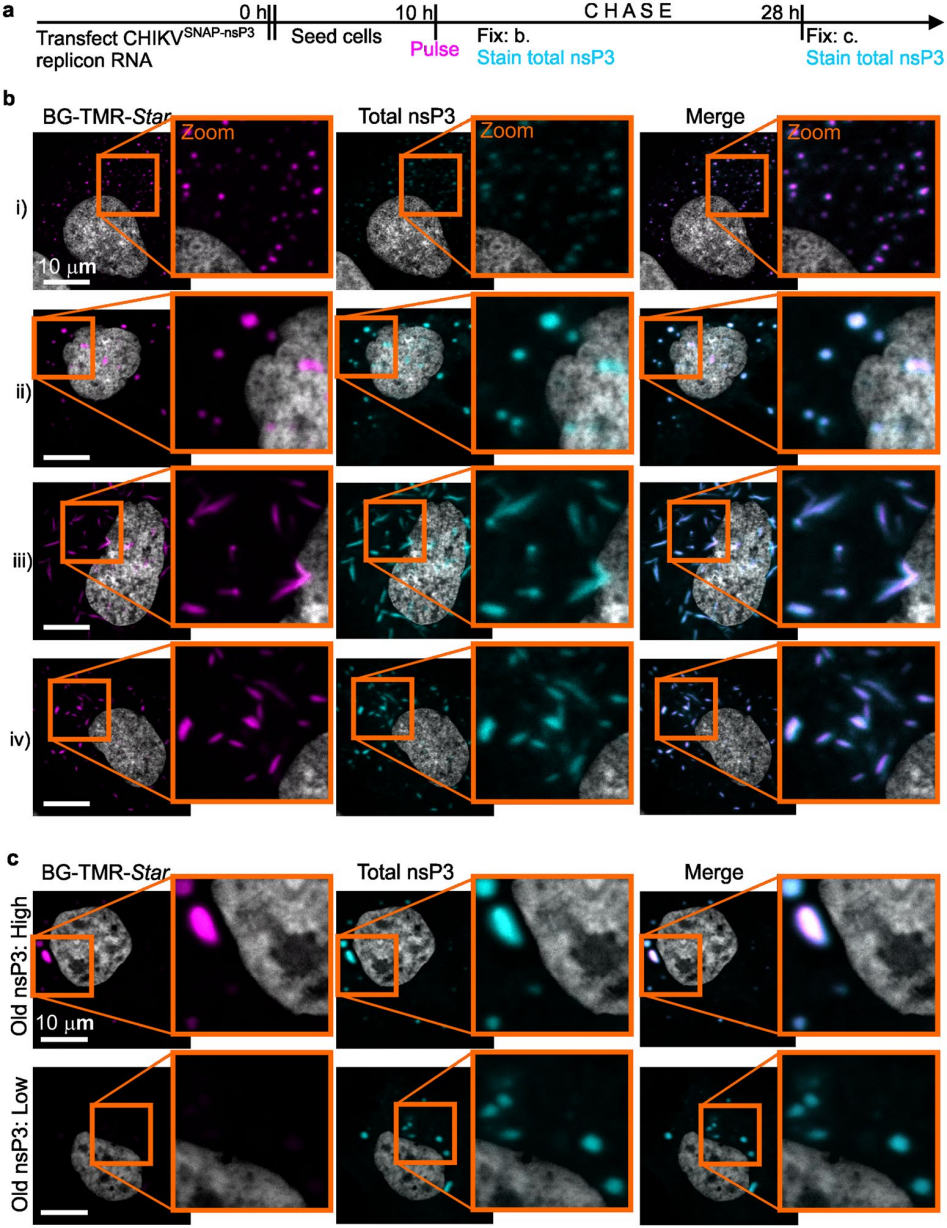
The appearance of SNAP-nsP3-positive structures is time-dependent and transient. (**a**) HuH-7 cells were transfected with CHIKV^SNAP-nsP3^ RNA and incubated with BG-TMR-*Star* (pulse) ten hours after transfection, turning the available pool of SNAP-nsP3 fluorescent. After washout of the substrate (chase), cells continued to synthesise SNAP-nsP3, which was not labelled, while the pulsed pool turned over. The remaining fluorescently labelled pool of SNAP-nsP3 was visualised after an 18-h chase by confocal microscopy. The total pool of nsP3 was detected with nsP3-specific antiserum. Data was acquired with a Zeiss LSM 880 confocal system with Airyscan, operated in the confocal mode with a Plan-Apochromat 63x oil objective (1.4 numerical aperture). (**b**) Images from pulse-chase experiment show cells fixed immediately following the pulse ten hours after transfection. Cells with the two main classes of nsP3 distributions, granules (i and ii) and rod-like structures (iii and iv) are shown. Insets are enlargements that show an overlap between SNAP-specific signal (pink) and antibody-specific signal (cyan). (**c**) Images from pulse-chase experiment show cells fixed after a pulse at ten hours after transfection, a chase media lacking BG-TMR-*Star* for 18 h, chemical fixation, and immunostaining for total nsP3. Cells were classified based on the level of BG-TMR-*Star* staining, which was high in some cells (upper panel) and low in others (lower panel). Representative cells are shown. Zoomed-in views of cells with a high level of SNAP-specific signal (pink) revealed that the signal from the total pool of nsP3 (cyan) co-localised. Zoomed-in views of cells with a low level of SNAP-specific signal (pink) indicated that the staining of total nsP3 (cyan) was present at a high level in the same structures, whereas the SNAP-specific signal was low but still detectable. Cells were counterstained with DAPI (grey) to visualise nuclei.

A benefit of the SNAP system is the temporal control of labelling within an experiment. Thus, we wanted to test whether a pulse-chase strategy could label temporally separated populations of SNAP-nsP3 in living cells. After initial labelling by the addition of fluorescent BG-TMR-*Star* (Pulse, at 10 h post-transfection) and washout of excess dye, cells were incubated for 18 h (Chase) in cell culture media lacking BG-TMR-*Star* (Fig. 5a). During this chase period the labelled, “old” protein pool underwent trafficking and protein turnover, while cells synthesised unlabelled, “new” protein pools. After chemical fixation at the end of the chase period, we probed the total pool of SNAP-nsP3 with nsP3-specific antiserum. Further analysis by confocal microscopy provided insight into SNAP-nsP3 protein dynamics as it took place over the course of the 18-hour-chase period.

At 10 h post-transfection and 0 h post-labelling, the distribution of SNAP-nsP3 varied in cells, which predominantly contained either small granules (Fig. 5b, i), large granules (Fig. 5b, ii), long rod-like structures (Fig. 5b, iii), or short rod-like structures (Fig. 5b, iv). The SNAP-specific fluorescent signal (equivalent to old nsP3) and fluorescent signal of the total nsP3 pool overlapped, indicating that no unlabelled, new nsP3 had been synthesised yet (Fig. 5b, white colour in merge panels). At 28 h post-transfection and 18 h post-labelling, we still detected a high level of SNAP-nsP3 in a population of cells (Fig. 5c, panel “Old nsP3: High”). Fluorescent signals from the old nsP3 pool overlapped completely with the total pool of nsP3. The absence of BG-TMR-*Star*-negative clusters suggested that new nsP3 did not accumulate at *de novo* sites. Also, the results could reflect a reduced synthesis of new nsP3 due to auto-shutdown of non-structural protein synthesis, as has been documented for alphaviruses. In another population of cells, we detected a low level of SNAP-specific fluorescent signal, while the total level of nsP3 remained high (panel “Old nsP3: Low”). This overlap was consistent with new nsP3 pools accumulating at existing sites already containing old pools of nsP3. Cell-to-cell differences in the level of old nsP3 (high versus low) could be the result of differences in protein turnover between cells or differences in the level of nsP3 during the initial pulse. Moreover, we could not rule out that the population of cells that lacked a high level of labelled nsP3 was the result of desynchronisation in the experiment, as alphavirus replicons can also spread from replicon-containing cells to naïve cells^55, 56^. Nonetheless, cells no longer contained small granules, long rods, or short rods after the pulse-chase; rather nsP3 was concentrated in large clusters (Fig. 5c, zoomed insets).

Through live-cell imaging of temporally labelled nsP3 (pulsed at 11 h), we could also monitor the dynamics of SNAP-nsP3 cluster formation and disassembly in real-time. Time-lapse imaging (one image per hour, starting 14 h after transfection and ending nine hours later) also revealed that cells with a granular appearance at 14 h retained the granular phenotype throughout the time-lapse series (Supplementary Fig. S2). However, cells that initially contained rod-like structures only contained large, granular structures towards the end of the time-lapse series (Supplementary Fig. S2). Together, the results from the pulse-fix, pulse-chase, and pulse-image experiments are consistent with an ongoing mixing of old and new nsP3-molecules in granular structures. Furthermore, the presence of rod-like nsP3 structures was time-dependent (not observed at later time points) and transient (with rod-like structures disassembling and forming larger clusters over time). Thus, the SNAP-tagged replicon opens up new avenues to investigate questions about protein trafficking and turnover.

### Granular and rod-like nsP3 structures are proximal to nsP1 structures

**Figure 6:**
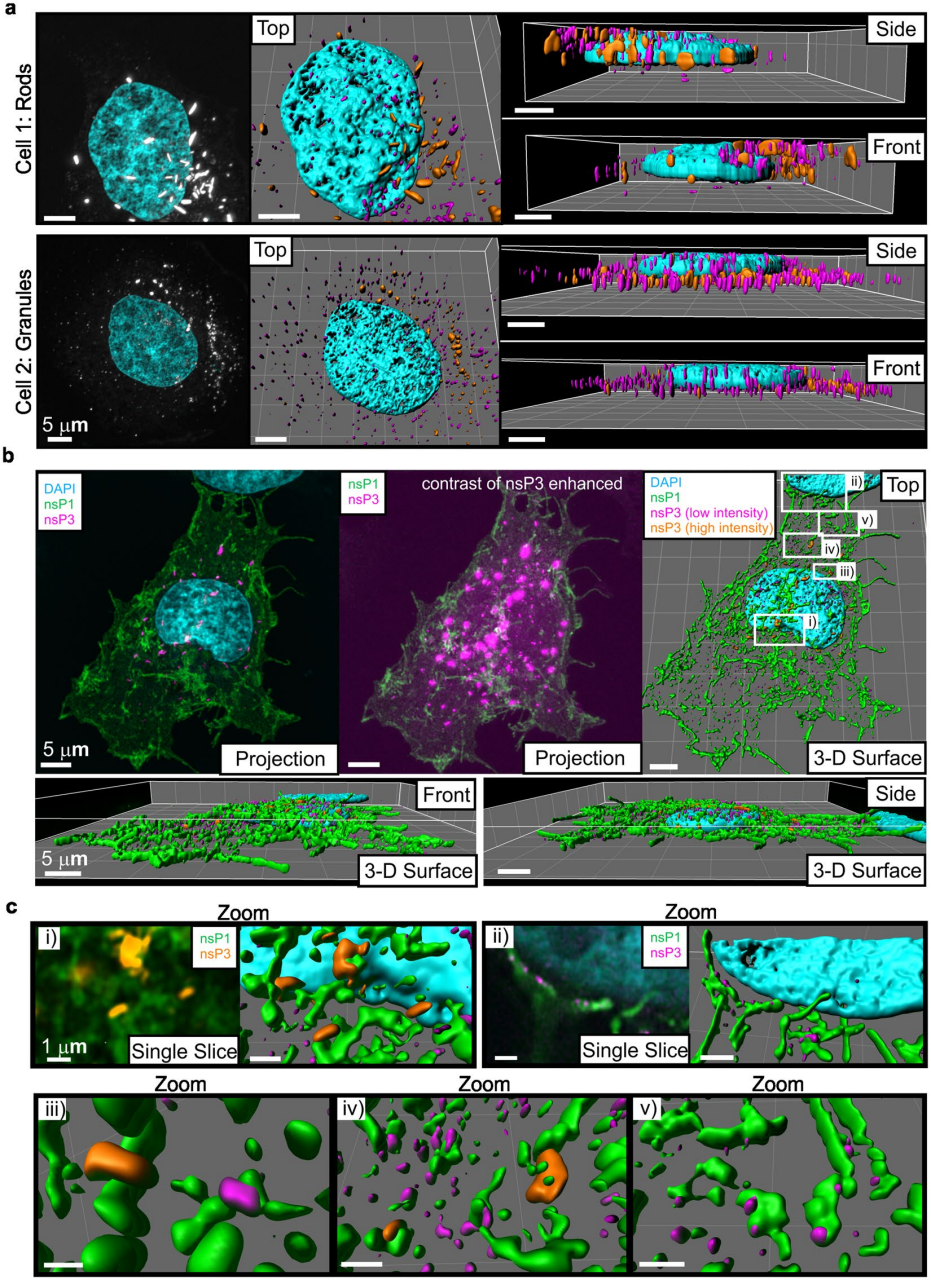
3-D visualisation of clusters of SNAP-nsP3 and proximity with nsP1-rich regions. HuH-7 cells were transfected with CHIKV^SNAP-P3 sg-ZsGreen^, fixed after 12 hours, labelled with BG-TMR-*Star* and imaged with a Zeiss LSM 880 confocal system with Airyscan, operated in the super-resolution mode. (**a**) Z-stacks consisted of 43 sections over an axial distance of 6.7 µm (Cell 1) or 27 sections over 5.2 µm (Cell 2). A lens power of 63× (1.4 numerical aperture) was used with a digital zoom factor of 4 (Cell 1) or 3 (Cell 2). Voxel sizes were 37 × 37 × 150 nm (Cell 1) or 37 × 37 × 200 nm (Cell 2). Images on the left represent a maximum-intensity projection of the BG-TMR-*Star* channel. Images on the right show surface views of the 3-D reconstruction from a cell (Cell 1) containing both rod-like structures and granular clusters. Two separate thresholds were applied during rendering of surfaces to visualise clusters with high-fluorescence-intensity (orange) and low-fluorescence-intensity (pink). Top, front and side views of the 3-D volumes are shown. Compared to cell 1, cell 2 contained more low-intensity, granular clusters. DAPI-stained cell nuclei are displayed in cyan. (**b**) Antibodies detecting nsP1 were used for immunostaining of HuH-7 cells that were prepared as in (**a**). Z-stack consisted of 20 sections over a total axial distance of 4.3 µm. A lens power of 63× was used with a digital zoom factor of 2.7 and voxel size of 37 × 37 × 224 nm. Maximum-intensity projections of the Z-stack displayed signals from BG-TMR-*Star* labelling (nsP3, pink), nsP1 immunostaining (green) and DAPI staining (cyan). Middle image represents a projection following digital processing of the nsP3 images to enhance the image contrast and reveal clusters with low-fluorescence-intensity. Surfaces of 3-D reconstructions were rendered as in (**a**). (**c**) Zoomed-in views of the boxed regions in (**a**) were labelled i-v. For comparison, single slices in volume rendering mode (with the regular contrast of the nsP3 images in orange and the enhanced contrast in pink) are shown alongside the images from the surface rendering mode (i and ii). All 3-D surfaces were created with Imaris software.

To visually assess the 3-D distribution of SNAP-nsP3 and uncover spatial relationships that are not apparent in 2-D analyses, we combined SNAP-based labelling, 3-D confocal super-resolution microscopy, and a volume-rendering software. A benefit of imaging with a confocal system with Airyscan technology is the microscope’s 3-D imaging capability. This approach confirmed that nsP3 distribution varied between cells. Whereas some cells contained elongated rod-like structures (Fig. 6a, Cell 1), other cells contained round, granular structures (Fig. 6a, Cell 2). In this study, we also refer to these structures as clusters, which were areas with a concentration of nsP3-specific fluorescence intensity. Clusters of nsP3 within each cell varied in their protein density. This variation complicated the fluorescence image acquisition because visualisation of lower-density clusters required a more sensitive detection set-up, which in turn overexposed the brighter, high-density clusters within the same field-of-view.

To visualise both low-density and high-density protein clusters, we imaged with detector settings that did not saturate pixels and then manually set two different thresholds during the 3-D reconstruction of the Z-stacks. First, we set a high threshold for high-density protein clusters such as rod-like structures and large granules. Second, we set a low threshold combined with a filter for fluorescence intensity; the second approach selectively displayed small clusters with low density. The resulting surface rendering contained both rod-like structures (Fig. 6a, orange) and small granules (Fig. 6a, pink) inside the same cell. In cells with a granular appearance, the thresholding approach separated high-density clusters from low-density clusters as well (Fig. 6a, orange and pink; Supplementary Videos S1 and S2 for Cell 1 and 2). Subsequently, we wanted to examine the relationship between these different forms of nsP3 and additional viral markers, such as the double-stranded RNA (dsRNA) replication intermediate. Small, nsP3-positive granules were associated with dsRNA foci at the top or bottom of cells (Supplementary Fig. S3), most likely at sites connected with the plasma membrane as other groups have shown previously^19, 27^. In contrast, the juxtaposition of dsRNA-foci and intracellular, rod-like clusters of nsP3 was infrequent (Supplementary Fig. S3). In conclusion, combining SNAP-labelling with 3-D microscopy and imaging analysis allowed us to visualise populations that varied between cells (rod-like structures vs. granular protein-clusters) but also within each cell (high-density vs. low-density protein clusters).

Next, we wanted to determine the relationship between nsP3-containing clusters and nsP1, a major component of viral replication complexes. Our protocol included SNAP-labelling of cells transfected with CHIKV^repl SNAP-P3 sg-ZsGreen^ RNA, chemical fixation, counterstaining with antiserum specific for nsP1, photon-pixel-reassignment microscopy with Airyscan, and processing of each deconvolved confocal laser-scanned dataset with a 3-D visualisation software. Staining for nsP1 and nsP3 was only seen in ZsGreen-positive cells (Figure 6b; an untransfected cell is shown at the top of the image and a transfected cell in the centre, note that ZsGreen-specific channel was hidden digitally to emphasise nsP1 and nsP3 staining). NsP1 was notably present in filopodia-like extensions at the edge of the cell (Fig. 6b) and was also distributed in a more reticular pattern inside the cell (Fig. 6b). Signals from the SNAP-specific and nsP1-specific labels did not overlap in the 3-D volume (Fig. 6b and Supplementary Video S3). This lack of co-localisation between nsP1 and nsP3, known to interact in co-immunoprecipitation assays, may be the result of an inability to detect lower concentrations of CHIKV nsP1 in replication organelles by immunofluorescence assays. Nonetheless, the high-resolution 3-D dataset provided insight into the relationship of nsP3-positive and nsP1-positive structures (Supplementary Video S3). Fluorescent signals from nsP1 appeared to form an interconnected network and nsP3 rods connected to this network at distinct points in the perinuclear region (Fig. 6b, i and iii). Low-density clusters of nsP3 were also found near perinuclear, nsP1-positive regions (Fig. 6b, iii). We observed similar associations in cytoplasmic regions further away from the nucleus and at the cell periphery (Fig. 6b, iv and v). Although the majority of filopodia-like extensions were only nsP1-positive and did not connect to clusters of nsP3 as they did in the perinuclear region, structures with a low density of nsP3 were found close to filopodia-like extensions in some cases (Fig. 6b, ii). Thus, examining the spatial relationships by high-resolution 3-D microscopy revealed that nsP1- and nsP3-positive regions were found adjacent to each other in 3-D space.

### Microscopy reveals proximity of nsP3-structures and lipid droplets in hepatic cells

**Figure 7:**
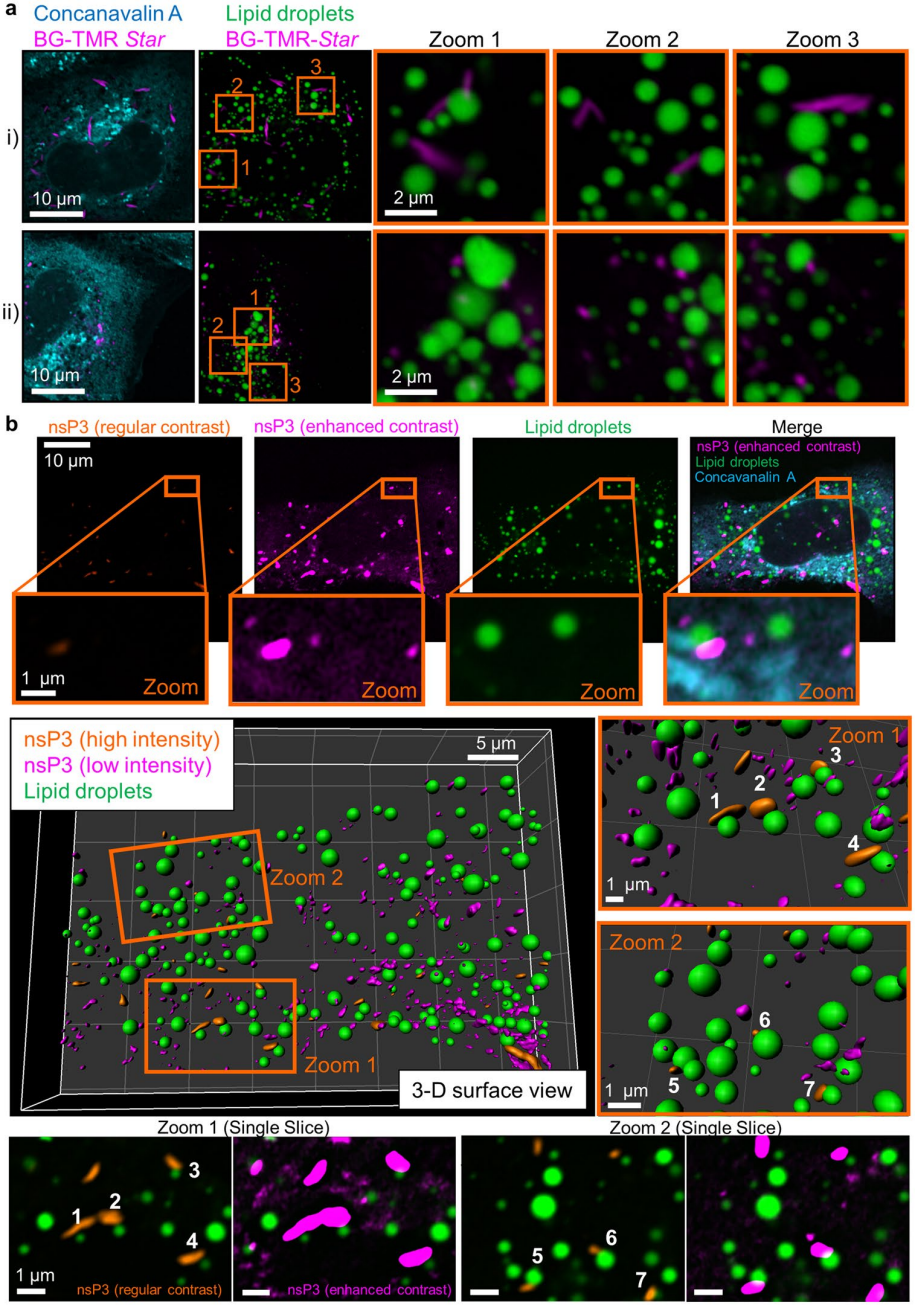
NsP3-positive structures are close to the surface of lipid droplets in hepatic cells. Monodansylpentane (MDH), which has a fluorescence emission between 420 and 480 nm when incorporated in lipid droplets, was used to stain lipid droplets (green). MDH was combined with the SNAP-specific red fluorescence of BG-TMR Star (magenta) and the cellular counterstain Concanavalin A conjugated to Alexa Fluor 647 dye (cyan), which selectively binds to α-mannopyranosyl and α-glucopyranosyl residues on cell membranes, such as ER and Golgi complex. (**a**) Cells with a predominance of rod-like structures (i) or granular structures (ii) were imaged with a Zeiss LSM 880 confocal system with Airyscan, operated in the super-resolution mode. Images show individual slices, which were extracted from the Z-stack of staining. Merged images of the BG-TMR-*Star* channel (pseudo-coloured in pink) and either Concanavalin A (cyan) or lipid droplets (green) are shown. Zoomed-in views are in the panels on the right (Zoom 1–3). (**b**) A single slice shows a region of interest from the sample prepared in (**a**). To illustrate the presence of low-intensity nsP3 clusters, we digitally enhanced the contrast of the nsP3-specific signal in one image (“enhanced contrast”). 3-D surfaces were generated from the 3-D reconstruction of Z-stacks, which consisted of 38 slices over an axial distance of 5.9 µm. Both surface rendering and volume rendering mode were chosen for zoom-ins of two intracellular regions (Zoom 1 and 2). SNAP-nsP3 rod-like structures (1–4) and granular structures (5–7) were located close to lipid droplets.

Many viruses interact with lipid metabolism pathways of their host, and cellular lipid droplets play key roles during the viral life cycle^57, 58^. HuH-7 cells are known to contain numerous lipid droplets, and purification of lipid-droplet-enriched fractions revealed the presence of multiple lipid-associated and lipid-metabolising proteins^59^. To our knowledge, whether alphaviral nsP3 proteins can associate with lipid droplets remains unknown. Therefore we wanted to examine the spatial relationship between lipid droplets and nsP3 by 3-D super-resolution microscopy. HuH-7 cells were fixed, labelled with BG-TMR-*Star* and stained with monodansylpentane (MDH), which preferentially fluoresces in the blue spectrum when incorporated in lipid droplets^60^. NsP3 did not co-localise with lipid droplets and did not coat their surfaces; in several examples, however, nsP3 was in the vicinity of lipid droplet surfaces (Fig. 7a). Examples of this proximity included both rod-like structures (Fig. 7a, i) and granular clusters (Fig. 7a, ii). High-resolution 3-D reconstructions also uncovered this proximity between the surface of lipid droplets and elongated, rod-like structures (Figs 7b, 1–4) as well as smaller granules (Figs 7b, 5–7). An animation of the 3-D rendered volumes further supports this association between nsP3 and lipid droplets (Supplementary Video S4). In conclusion, the super-resolution 3-D microscopy data provided evidence that nsP3-positive structures are located close to cytoplasmic lipid droplets in human hepatic cells.

## Discussion

Here, we expand the panel of fluorescent CHIKV derivatives by generating a replicon that encodes SNAP-tagged nsP3. We characterised functional properties of the tagged replicon in human cells and showed its versatility for studying the morphology and distribution of nsP3-positive structures, the turnover of SNAP-nsP3 over time, the spatial relationship between nsP1 and nsP3, and the association of nsP3 with lipid droplets. SNAP-nsP3 formed rod-like structures, which disassemble over time. By developing protocols for the visualisation of SNAP-nsP3 that are compatible with recently developed imaging technologies such as Airyscan super-resolution confocal imaging, we lay the foundation for more detailed studies of nsP3 function within the cellular host environment.

Tagging of non-structural proteins with fluorescent proteins has already been successful for many viruses, including poliovirus, hepatitis C virus, equine arteritis virus, and filamentous plant viruses^61–64^, among others. Also, various research groups have developed fluorescent derivatives of CHIKV^31, 40, 41, 43, 65, 66^, but none of these constructs contained the SNAP polypeptide. Advantages of SNAP-tagged derivatives of human immunodeficiency virus (HIV) over existing fluorescent protein-based constructs include: 1) the ability to pick different dyes according to specific needs in multicolour applications 2) compatibility with sequential labelling protocols in pulse-chase experiments 3) the possibility to label SNAP-tagged protein with synthetic dyes that have been validated for super-resolution microscopy techniques^67^. For the SNAP-tagged CHIKV replicon, we confirmed that SNAP-nsP3 could be used for pulse-chase imaging, which allows for the analysis of protein turnover. We predict that these replicons will also be useful for (live-cell) super-resolution microscopy of SNAP-tagged proteins in approaches that use stochastic blinking^68, 69^. Finally, additional applications of SNAP-based systems could complement the microscopy-based methods presented here. For example, the SNAP technique has been useful in proteomic pull-down studies^70–72^ and for *in vivo* imaging of small animals^46, 73–75^.

Although the SNAP polypeptide is shorter than typical fluorescent proteins (by about 1/3), its fusion to nsP3 still had an effect on viral replication. This effect may be related to non-structural polyprotein processing and the larger overall size of SNAP-nsP3 compared to untagged nsP3. Delays or defects in the formation of replication complexes are also possible, as well as the disruption of viral RNA structural elements by the introduction of foreign sequences. Tagging with fluorescent proteins also attenuated replication and growth in previous studies of alphaviruses^31, 42, 65^. Despite this reduction in replicative capacity, the SNAP-tagged CHIKV replicons still retained known functional properties, such as the nsP3:G3BP interaction, the formation of granular and rod-like nsP3-positive structures, and a block of arsenite-induced stress granule responses. Moreover, fluorescence microscopy revealed a concentration of dsRNA foci at the cell periphery (which was previously described for untagged nsP3^19, 27^) and the formation of nsP1-positive filopodia-like extensions (as seen for SINV and SFV^76^). Thus, the reduction in replicative capacity is unlikely to preclude future use of the SNAP-tagged replicon, but for each new application of this replicon, it is advisable to verify that the presence of the SNAP-tag does not affect the specific nsP3 functions under investigation.

A characteristic feature of SNAP-nsP3 subcellular distribution in HuH-7 cells was the presence of rod-like structures. Various studies reported the formation of filamentous structures (Supplementary Text S1). SNAP-nsP3 rod-like structures resemble the G3BP-positive structures induced upon CHIKV infection of Vero E6 cells^37^. Rod-like structures in HuH-7 cells contained a high level of G3BP proteins but did not sequester eIF4G in a similar fashion. Thus, SNAP-nsP3 appears to retain the ability to sequester G3BP in either granules or rod-like structures and block the formation of genuine (eIF4G-positive) stress granules. Formation of rod-like structures was transient; at later points of the viral replicative cycle, cells contained large granular clusters of nsP3 instead. Since the SNAP-system is compatible with live-cell imaging, future studies may examine the exact times at which times different forms of nsP3 (found in rod-like, granular, high-density, or low-density clusters) appear following transfection of SNAP-tagged replicons. Moreover, live-cell imaging will allow researchers to track the different forms of nsP3 over time and correlate changes in nsP3 distribution with specific time points. Lastly, our pilot study only examined SNAP-nsP3 distribution in hepatic cells and the replicon system, but additional studies are ongoing to determine whether SNAP-nsP3 distribution patterns are conserved in human, mammalian, or insect cells and whether virus constructs containing SNAP-nsP3 retain the ability to produce infectious virus particles.

In addition to the improvement in nsP3 labelling, this study also advances the detection and visualisation of nsP3 by combining SNAP-tag labelling with high-resolution light microscopy techniques, such as Airyscan confocal super-resolution microscopy (the advantages of pairing SNAP-labelling, Airyscan microscopy, and 3-D processing software are further discussed in Supplementary Text S1). With this approach, we could visualise nsP3-positive clusters with high and low density within the same cell. The appearance of these different forms of CHIKV nsP3-positive structures is consistent with the participation of SINV nsP3 in various complexes in infected cells^33^ and is likely to relate to the multiple roles that the nsP3 protein carries out during the viral life cycle. Much remains to be learned about the exact structure and morphology of the different clusters of CHIKV nsP3 shown in this study. The SNAP-tagged version of nsP3 can be particularly useful in this endeavour, as a SNAP-tagged version of hepatitis C virus NS5A has recently been used for correlative light and electron microscopy to characterise the ultrastructure of different replication organelles^77^.

This study also provides new insight into the spatial relationships between nsP3 and nsP1. Alphaviral nsP1 is known to have an intrinsic ability for membrane binding^16, 78^ and induces filopodia-like extensions during SINV and SFV infection^76^. NsP1 may bind non-structural proteins such as nsP3 in replication complexes and tether these complexes to cellular membranes through its membrane binding domain^79^. SFV nsP1 co-localises with G3BPs and dsRNA, presumably in replication complexes^36^. However, in Vero cells infected with CHIKV, signals for G3BP2 and nsP1 did not overlap, and only partial co-localisation was detected between G3BP2 and dsRNA at early times post-infection^37^. Since nsP3 distribution was shown to overlap with G3BP2 distribution in the aforementioned study, we would expect nsP1 and nsP3 not to co-localise under the experimental conditions of Scholte *et al*.; thus, the lack of nsP1/nsP3 co-localisation may be a consequence of the inherent detection limits of immunofluorescence compared to co-immunoprecipitation methods, which previously demonstrated an interaction between these two replication complex components. Nonetheless, our 3-D microscopy results build upon the existing data on the nsP3-nsP1 interaction by examining the spatial relationship between these two proteins in more detail. Whereas nsP3 was organised in different types of free clusters (with both granular and rod-like morphology), nsP1 was found in an interconnected intracellular network, in detached protein clusters, or on the plasma membrane. We also confirmed that nsP1 formed filopodia-like extensions in hepatic cells and showed various examples of proximity between nsP1- and nsP3-rich regions. Thus, although nsP3 did not co-localise with nsP1, nsP1- and nsP3-positive clusters viewed in three dimensions were clearly organised in a way that placed them in the same vicinity. Thus, it is likely that some of these neighbouring structures that had high concentrations of nsP1 and nsP3 represent viral replication complexes. Our labelling protocol can form the basis for future 3-D multicolour microscopy approaches that evaluate the nsP3-nsP1 relationship in the context of additional viral markers (dsRNA, nsP2, nsP4) or cellular markers (such as G3BP, or markers for endosomes, lysosomes, mitochondria, ER and Golgi complex).

In addition to examining the association between nsP3 and nsP1, we also tested whether nsP3 was localised close to lipid droplets in HuH-7 cells. Lipid droplets are dynamic organelles that store intracellular lipid reserves and play roles in cellular lipid homoeostasis^80–82^. Recruitment of viral proteins to lipid droplet associated membranes of HuH-7 cells is critical for producing infectious hepatitis C virus^83^. In this study, 3-D microscopy of SNAP-nsP3 and lipid droplets provided several examples in which nsP3 was located close to the surface of lipid droplets. To our knowledge, this is the first study reporting proximity between nsP3 and lipid droplets. Further studies will be needed to determine whether this association is essential for viral replication, whether the presence of CHIKV proteins has any effects on the lipid droplet microenvironment, and to define the composition of the lipid droplet microenvironment in CHIKV-positive cells. Such studies will help to shed further light on the relationship between lipid metabolism and alphaviral replication, which has not been extensively investigated, though a few studies point towards an important connection (Supplementary Text S1).

In conclusion, we have presented a SNAP-tagged derivative of CHIKV as a new tool for the visualisation of nsP3 during the CHIKV replicative cycle. Protocols described here for the 3-D analysis of nsP3-clusters and multicolour staining with various markers (nsP1, stress granules, lipid droplets) improve existing methods to analyse and track nsP3. The great versatility of the SNAP-tagged replicon with regards to microscopy applications will benefit functional studies of nsP3 to further uncover its role during viral infection, for example by 3-D imaging of nsP3 and its interacting partners in living host cells over time. The lack of a licensed vaccine or approved antiviral drugs against CHIKV necessitates a better understanding of its viral life cycle so that essential steps can be targeted therapeutically in the future.

## Methods

### Plasmids, cell culture, and transfection of replicon RNA constructs

#### Construction of DNA plasmids containing CHIKV replicons

DNA constructs containing the CHIKV-replicon cDNA from the LR2006 OPY1 strain, which was originally isolated from the serum of a febrile patient travelling from La Réunion^65^, with or without ZsGreen in the region 3′ of the subgenomic promoter, were constructed previously^41, 43^ and are referred to as CHIKV^repl^ and CHIKV^repl sg-ZsGreen^. Briefly, cDNA fragments (Geneart) were synthesised based on the published sequence of the LR2006 OPY1 strain and assembled *in vitro* to generate these fully synthetic replicons. Also, a CHIKV replicon containing the mCherry gene fused to nsP3, referred to as CHIKV^repl mCherry-P3^ was used as the starting material for cloning of a SNAP-tagged replicon. In this construct, the mCherry gene (Clontech), is between two unique SpeI restriction sites, which were previously introduced into the CHIKV genome. CHIKV^repl mCherry-P3 sg-ZsGreen^ was generated by inserting a ZsGreen-containing DNA fragment (derived from CHIKV^repl sg-ZsGreen^) into CHIKV^repl mCherry-P3^ after digestion with AvrII and NotI restriction enzymes, purification of the insert and vector DNA, and ligation with T4 DNA ligase. The SNAP sequence was produced by PCR amplification of the pSNAP_f_ vector (New England Biolabs, NEB) with primers containing SpeI sites and a sequence encoding short amino acid linkers (5′-GCC ATC CAC AAC TAG TGG TGG CGG CAT GGA CAA AGA CTG CGA AAT GAA-3′ and 5′-GCA AGG TTT CCA CTA GTC CCT CCA CCC AGC CCA GGC TTG CCC A-3′). The purified PCR product was used to replace mCherry from the CHIKV^repl mCherry-P3 sg-ZsGreen^ construct by restriction digestion and ligation of the SpeI sites. To generate a construct without any reporter genes 3′ of nsP4 (CHIKV^repl SNAP-P3^), we digested a CHIKV^repl SNAP-P3 sg-ZsGreen^ DNA plasmid with AvrII and PmeI, treated with DNA polymerase I (Large [Klenow] Fragment) or Mung bean nuclease to blunt DNA ends, followed by re-ligation of the blunted plasmid. All constructs were verified by DNA sequencing of nsP3 regions and the subgenomic region, as well as analysis of EcoRI/BamHI restriction digest patterns to test for the overall integrity of CHIKV replicons.

#### Cell culture

HuH-7 is a well differentiated hepatocyte-derived cellular carcinoma cell line that was originally taken from a liver tumour in a 57-year-old Japanese male in 1982^84^; these cells were obtained from John McLauchlan (Centre for Virus Research, Glasgow). HuH-7 cells were maintained in complete media, which consisted of DMEM (Dulbecco’s modified Eagle’s medium) supplemented with 10% fetal calf serum, 100 IU penicillin/ml, 100 μg streptomycin/ml, a 1/100 dilution of MEM (Minimum Essential Media) Non-Essential Amino Acids Solution, and 10 mM HEPES Buffer. Cells were kept in a humidified incubator at 37 °C with 5% CO_2_. For live-cell experiments, the above supplements were added to modified DMEM with high glucose and without phenol red, L-glutamine, and sodium pyruvate. Where indicated, cells were stressed by the addition of sodium arsenite for 30–45 min, diluted to a final concentration of 1 mM in complete media.

#### *In vitro* transcription and transfection of replicon constructs

Plasmids containing cDNA of CHIKV replicons were linearised by NotI digestion. Purified DNA was used as the template for an *in vitro* transcription reaction based on the mMESSAGE mMACHINE SP6 transcription kit (Ambion). RNA was column-purified and kept in aliquots of distilled H_2_O at −80 °C until the day of transfection. For live-cell experiments with the IncuCyte system, cells seeded one day before transfection in 24-well plates were transfected by Lipofectamine 2000 (Thermo Fisher Scientific) according to manufacturer’s instructions. For all other experiments, replicon RNA was electroporated by harvesting cells at 70–90% confluency from 10-cm dishes by trypsinisation, resuspending in 550 µl Opti-MEM Reduced Serum Media (Gibco), and electroporating in 0.4 cm-wide microcuvettes with 2–5 μg of *in vitro* transcribed RNA and an exponential decay protocol set at 260 V and 950 μF capacitance. Subsequently, cells were resuspended in complete DMEM and seeded in 24-well plates for fixed-cell experiments with the IncuCyte system, 24-well plates containing No. 1.5 round glass coverslips with a 12 mm diameter for confocal microscopy, or in 2-well μ-Slides with a No. 1.5 polymer coverslip bottom (Ibidi) for live-cell confocal microscopy.

### Fluorescent staining and microscopy

#### Cellular stains and antibodies used in fluorescence microscopy of fixed cells

For experimental detail on staining dyes and antibodies used for immunofluorescence microscopy, see Supplementary Text S1.

#### Quantification of replicon-driven ZsGreen-expression

ZsGreen expression was quantified with an IncuCyte ZOOM system (Essen BioScience), which consists of an automated phase-contrast and fluorescent microscope housed within a humidifying incubator connected to a 5% CO_2_ line. For a detailed description, see Supplementary Text S1.

#### Intracellular SNAP-tag labelling and pulse-chase experiments

HuH-7 cells were transfected with *in vitro* transcribed RNA of CHIKV^repl SNAP-P3^ to express SNAP-tagged nsP3. Transfected cells were then plated in 24-well plates containing glass coverslips. SNAP-nsP3 was fluorescently labelled as described in Bodor *et al*.^47^. Briefly, for immunofluorescence assays combined with SNAP-tag labelling, CHIKV^repl SNAP-P3^-transfected cells were fixed with 4% formaldehyde in PBS for 30 min at room temperature, followed by a minimum of three washes with phosphate buffered saline (PBS). TMR-*Star*-labelled benzylguanine (BG-TMR-*Star*, sold as SNAP-cell TMR-*Star*, NEB) was diluted to 1 µM final concentration in complete media and added to fixed cells for 15 min at 37 °C. For live-cell staining or pulse-chase experiments, BG-TMR-*Star* was added to live cells and incubated for 15 min at 37 °C, 5% CO_2_. To remove background fluorescence, we washed cells twice with complete media and washed a third time for a minimum of 30 min, according to a published protocol^47^. For pulse-chase experiments, 1 ml of complete media was re-added before incubating cells in standard growth conditions for the chase period (18 h), after which cells were fixed. Fixed cells stained with BG-TMR-*Star* were processed for immunofluorescence with the nsP3 antibody as described below.

#### Immunofluorescence assays and standard confocal microscopy

Experimental details of sample preparation for standard confocal microscopy can be found in the Supplemental Text S1. Immunofluorescence images were obtained and analysed with an LSM700 or LSM880 confocal microscope (Zeiss), equipped with Gallium Arsenide Phosphide (GaAsP) detectors and operated in the standard confocal mode. A Plan-Apochromat 63×/1.4 Oil Ph3 M27 objective was used for all experiments. All imaging channels were set up in the Zen Black software (Zeiss) with the ‘smart setup’ function, which generated separate image tracks to minimise cross-talk between channels. Pinholes were set to 1 Airy Unit for the longest emission channel, then matched according to thickness for the remaining tracks. Where necessary, averaging was set to up to four iterations in Line Mode (Mean Method) to reduce image noise. Pixel sizes and Z-stack intervals were set to the optimal values within the Zen software.

#### Sub-diffraction light microscopy

An LSM880 (Zeiss) upright confocal microscope with Airyscan was used to acquire sub-diffraction microscopy images. According to the system’s specifications, this microscope provides a lateral resolution of 140 nm and axial resolution of 400 nm for a fluorophore emitting at 480 nm. In the Airyscan super-resolution mode, complete airy disks were imaged onto the GaAsP detector array, which consisted of an array of 32 single detector elements arranged in a hexagonal pattern each representing 0.2 Airy units. A series of axial images (Z-stack) were acquired with a 63× 1.4 Numerical Aperture Plan Apochromat oil objective. To reassign the signals from the 32 detector elements to their correct position and obtain images with increased signal-to-noise ratio and resolution, we processed the image stacks with the Airyscan batch processing function of the Zen Black software.

#### 3-D reconstructions and analysis of microscope images

Imaris software (Version 8.3.0, Bitplane AG) was used to reconstruct 3-D data and obtain surface renderings of the signals in each fluorescent channel. To create surface renderings of dsRNA foci or lipid droplets, we used the software’s spot function. The co-localisation module was used to obtain scatterplots of voxels from multicolour imaging and to calculate Pearson’s correlation coefficients. Digital images were processed in Icy software^85^ to pseudo-colour imaging channels, adjust contrast and create zoomed-in views in Figs 4, 5, 6b and 7a,b.

#### Data availability

Image files and replicon constructs generated during and analysed during the current study are available from the corresponding author on reasonable request.

## Electronic supplementary material

Supplementary Information

Supplementary Video S1

Supplementary Video S2

Supplementary Video S3

Supplementary Video S4
