# Supplementary material for: SNAP-tagged Chikungunya Virus Replicons Improve Visualisation of Non-Structural Protein 3 by Fluorescence Microscopy
Source: Sci Rep. 2017 Jul 18;7:5682. doi: 10.1038/s41598-017-05820-0 (PMC5515888; doi:10.1038/s41598-017-05820-0)
Supplement: Supplementary file 1 — Supplementary Information [file 41598_2017_5820_MOESM1_ESM.pdf]

SNAP-tagged Chikungunya Virus Replicons Improve Visualisation of Non-Structural Protein 3 by Fluorescence Microscopy

Tagged nsP3 replicons for CHIKV research

Roland Remenyi[1], Grace C. Roberts[1], Carsten Zothner[1], Andres Merits[3], Mark Harris[1 2*]

Supplementary Information

Supplementary Text S1

Provides additional details on the Choice of HuH-7 cell line, the formation of filamentous structures during alphavirus replication, the combination of Airyscan microscope and 3-D surface rendering, links between lipid metabolism and alphavirus replication, cellular stains and antibodies used in fluorescence microscopy of fixed cells, immunofluorescence assays for standard confocal microscopy, and quantification of replicon-driven ZsGreen-expression.

Choice of HuH-7 cell line. As an experimental cell culture system, the well-characterized HuH-7 cell line offered several benefits for microscopy studies, such as a favourable nuclear-cytoplasmic ratio, a thinness of 6-10 μm (estimated in formaldehyde-fixed cells), and good adherence on glass coverslips, which were used in confocal microscopy. HuH-7 cells have already been used in high-throughput screening assays for the identification of pharmacological inhibitors against CHIKV (D.J. Cruz, R.M. Bonotto, R.G. Gomes, C.T. da Silva, J.B. Taniguchi, J.H. No, B. Lombardot, O. Schwartz, M.A. Hansen, L.H. Freitas-Junior, PLoS Negl Trop Dis 7: e2471, 2013). Also, liver tissues of infected mice contained nuclei inclusions of necrotic hepatocytes indicative of CHIKV-induced morphological damage (S. Lam, H. Chen, C.K. Chen, N. Min, J.J. Chu, Sci Rep 5: 12727, 2015). Together, these findings suggest that HuH-7 cells provide a cell-culture model that is relevant to the biology of CHIKV infection. Moreover, to our knowledge, a detailed characterization of CHIKV nsP3 subcellular localisation in human hepatic cells has been lacking.

Formation of filamentous nsP3 structures during alphavirus replication. Truncation of ten C-terminal amino acid residues from SFV-encoded nsP3 and expression of the mutant protein in BHK-21 cells results in the formation of filamentous nsP3-positive structures around the nuclei while also repressing the establishment of infection in HEK293 cells (M. Varjak, E. Zusinaite, A. Merits, J Virol 84: 2352-2364, 2010. Similarly, the complete deletion of nsP3's C-terminal domain in an EGFP-tagged CHIKV expression construct results in the formation of filaments instead of foci (J.J. Fros, N.E. Domeradzka, J. Baggen, C. Geertsema, J. Flipse, J.M. Vlak, G.P. Pijlman, J Virol 86: 10873-10879, 2012). Filaments in the study by Fros et al. did not co-localize with actin or tubulin, which are cytoskeletal markers, and thus oligomerization appears to be an intrinsic property of the conserved domain of alphaviral nsP3. Transient expression of nsP3 mutant proteins lacking the C-terminal domain in insect cells also resulted in the formation of filamentous, cytoplasmic structures (J. J. Fros, C. Geertsema, K. Zouache, J. Baggen, N. Domeradzka, D.M. van Leeuwen, J. Flipse, J.M. Vlak, A.B. Failloux, G. P. Pijlman, Parasit Vectors 8: 464, 2015). However, the nsP3-positive filaments described in the above studies differ from the nsP3-positive rods described in this study, since SNAP-nsP3 rods rarely formed long filaments as seen with truncation mutants.

Combination of Airyscan microscope and 3-D surface rendering. Airyscan confocal super-resolution microscopy was used to evaluate three-dimensional morphological features below the diffraction limit of light using a straightforward work flow. The optical sectioning capabilities of this system allowed us to acquire high-quality series of optical slices and reconstruct 3-D volumes from these image stacks. We found that the entire

depth of fixed HuH-7 cells could be captured by a series of 30-40 images, with individual acquisition times of 5-20 s for each image, depending on the number of channels imaged. The resulting images of SNAP-labeled nsP3 were high in contrast and could be processed within minutes using a commercial software for 3-D image processing and analysis. Visualisation of these structures by fluorescence microscopy can be technically challenging due to the large difference in fluorescence intensity between high-density clusters, such as fiber-like structures and low-density clusters, such as small granules. Using the thresholding functions of 3-D processing software, we have been able to visualise the surfaces of nsP3-positive clusters that have both high and low protein density.

Links between lipid metabolism and alphavirus replication. Previously, treatment with inhibitors of various lipid biosynthesis pathways reduced RNA replication of Semliki Forest virus (L. Perez, R. Guinea, L. Carrasco, Virology 183: 74-82, 1991) and CHIKV in vitro (A. Karlas, S. Berre, T. Couderc, M. Varjak, P. Braun, M. Meyer, N. Gangneux, L. Karo-Astover, F. Weege, M. Raftery, G. Schonrich, U. Klemm, A. Wurzlbauer, F. Bracher, A. Merits, T. F. Meyer, M. Lecuit, Nat Commun 7: 11320, 2016). In the study by Karlas et al., the chemical compound 5-tetradecyloxy-2-furoic acid, an inhibitor of acetyl CoA carboxylase, also had an antiviral effect in mouse models of CHIKV infection. Moreover, Semliki Forest virus replication complexes co-purify with fatty acid synthase and ATP citrate lyase, enzymes that play important roles in lipid biosynthesis (M. Varjak, S. Saul, L. Arike, A. Lulla, L. Peil, A. Merits, J Virol 87: 10295-10312, 2013).

Cellular stains and antibodies used in fluorescence microscopy of fixed cells. Cell organelles were stained in the following way: 4′,6-diamidino-2-phenylindole (DAPI) for

nuclei, the commercial version of monodansylpentane, AUTOdot Autophagy Visualisation Dye (Abgent), which stains lipid droplets, Alexa Fluor 647 Concanavalin A (Thermo Fisher Scientific), which selectively binds to alpha-mannopyranosyl and alpha-glucopyranosyl residues on cell membranes, for ER and Golgi complex. As the primary antibody in indirect immunofluorescence assays, we used the following commercial antibodies: monoclonal mouse anti-G3BP1 (Clone 23/G3BP, BD Bioscience), polyclonal rabbit anti-eIF4G (Clone H-300, Santa Cruz), polyclonal rabbit anti-G3BP2 (Bethyl Laboratories), monoclonal anti-dsRNA (J2, Scicons). Polyclonal antibodies against nsP1 and nsP3 were produced in-house (Merits laboratory). Whole species-specific IgG secondary antibodies (Thermo Fisher Scientific) were either anti-rabbit Alexa Fluor 633-conjugated IgG (to detect SNAP-nsP3 in Fig. 1 and 5, eIF4G and G3BP2 in Fig. 4, nsP1 in Fig. 6b,), anti-mouse Alexa Fluor 647-conjugated IgG (to detect G3BP1 in Fig. 4). Note that Mitotracker Green FM (Thermo Fisher Scientific) was used as a cellular counterstain for long-term tracking of live cells by confocal microscopy.

Immunofluorescence assays for standard confocal microscopy. At the indicated times (12 h, 28 h post-transfection) cells were washed with PBS and fixed with 4% formaldehyde in PBS. For analysis of stress granule markers or staining of dsRNA, cells were then permeabilised by incubating with 100% methanol at -20°C. Cells were then blocked for 30-60 min in a buffer containing 10% fetal calf serum in PBS. For staining of nsP1 and nsP3, cells were permeabilised by also including 0.1% Triton X-100 in this blocking buffer. Cells were then washed with PBS and stained with the aforementioned primary antibodies overnight in an antibody dilution buffer containing 5% bovine serum albumin in PBS (with dilutions of 1:1000 for nsP1 and nsP3, 1:200 for J2, 1:100 for

stress granule markers). Triton X-100 was added at 0.1% to the antibody dilution buffer that was added to cells that had already been permeabilised with Triton X-100, but left out in cells that had been permeabilised with methanol to avoid additional extraction of proteins in methanol-permeabilised cells. Primary antibody incubation was followed by a minimum of three washes in PBS and a 1-hour-incubation with dye-conjugated secondary antibodies in antibody dilution buffer. Where indicated, cells were counterstained with fluorescent dyes for nuclei (DAPI), lipid droplets (monodansylpentane), or α-mannopyranosyl and α-glucopyranosyl residues (concanavalin A), which are found in mammalian cell membranes, as described in Supplementary Text S1. Coverslips were mounted onto glass slides by the addition of ProLong Diamond Antifade Mountant (Thermo Fisher Scientific).

Quantification of replicon-driven ZsGreen-expression. Lipofection was used to transfect cells, which had been seeded in triplicate into 24-wells, with ZsGreen-containing replicons CHIKV$^{\text{repl sg-ZsGreen}}$, CHIKV$^{\text{repl SNAP-P3 sg-ZsGreen}}$, and CHIKV$^{\text{repl mCherry-P3 sg-ZsGreen}}$. Untransfected cells were included as a negative control. For live-cell studies, we captured microscopy images at hourly intervals for a total time of 20h starting at 7h post-transfection. Images were acquired using an IncuCyte ZOOM system (Essen BioScience), which consists of an automated phase-contrast and fluorescent microscope housed within a humidifying incubator connected to a 5% CO_2 line. In each well, the system was set to take nine images with the following software settings: Nikon 10x objective, dual-colour-filter module (model 4459), two image channels (Green and Phase with 1392 x 1040 pixels at 1.22 µm per pixel, acquisition time of 400 ms and 1000 ms respectively). Various metrics including overall cell confluency, green object

count (per mm^2), green object confluence (in %), mean fluorescence intensity (the green object's mean fluorescent intensity, in green calibrated units [GCU]) were analysed with the basic analyser module of the IncuCyte Zoom software. Graphed values are based on the mean values in each well, and standard error of measurement was calculated using the mean values from the triplicate wells for each replicon.

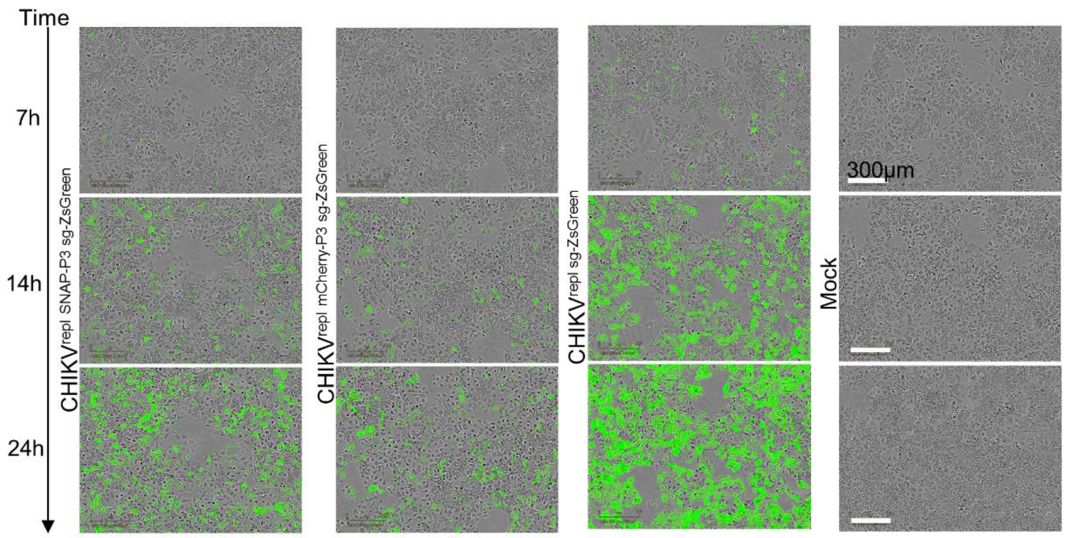

Supplementary Fig. S1 Green fluorescence as a read-out for replication of tagged replicons. Replication of tagged replicons in the background of CHIKV$^{\text{repl sg-ZsGreen}}$ in HuH-7 cells. After seeding HuH-7 cells into 12-well plates and an overnight incubation period to allow cells to adhere, we used lipofection to transfect CHIKV replicon RNA. We compared replication of CHIKV$^{\text{SNAP-P3 sg-ZsGreen}}$ (SNAP) and CHIKV$^{\text{mCherry-P3 sg-ZsGreen}}$ (mCherry) to that of CHIKV$^{\text{repl sg-ZsGreen}}$ (WT), which encodes untagged, wild-type nsP3. Negative controls of untransfected cells (Mock), which remained non-fluorescent throughout the time series, were included during the acquisition of images. Levels of ZsGreen protein were monitored hourly with an IncuCyte ZOOM live-cell analysis system, from 7 h until 27 h after transfection. Representative images show the same field-of-view at 7 h, 14 h, and 24 h post-transfection. Green signal from ZsGreen is presented in a merged view with phase-contrast images.

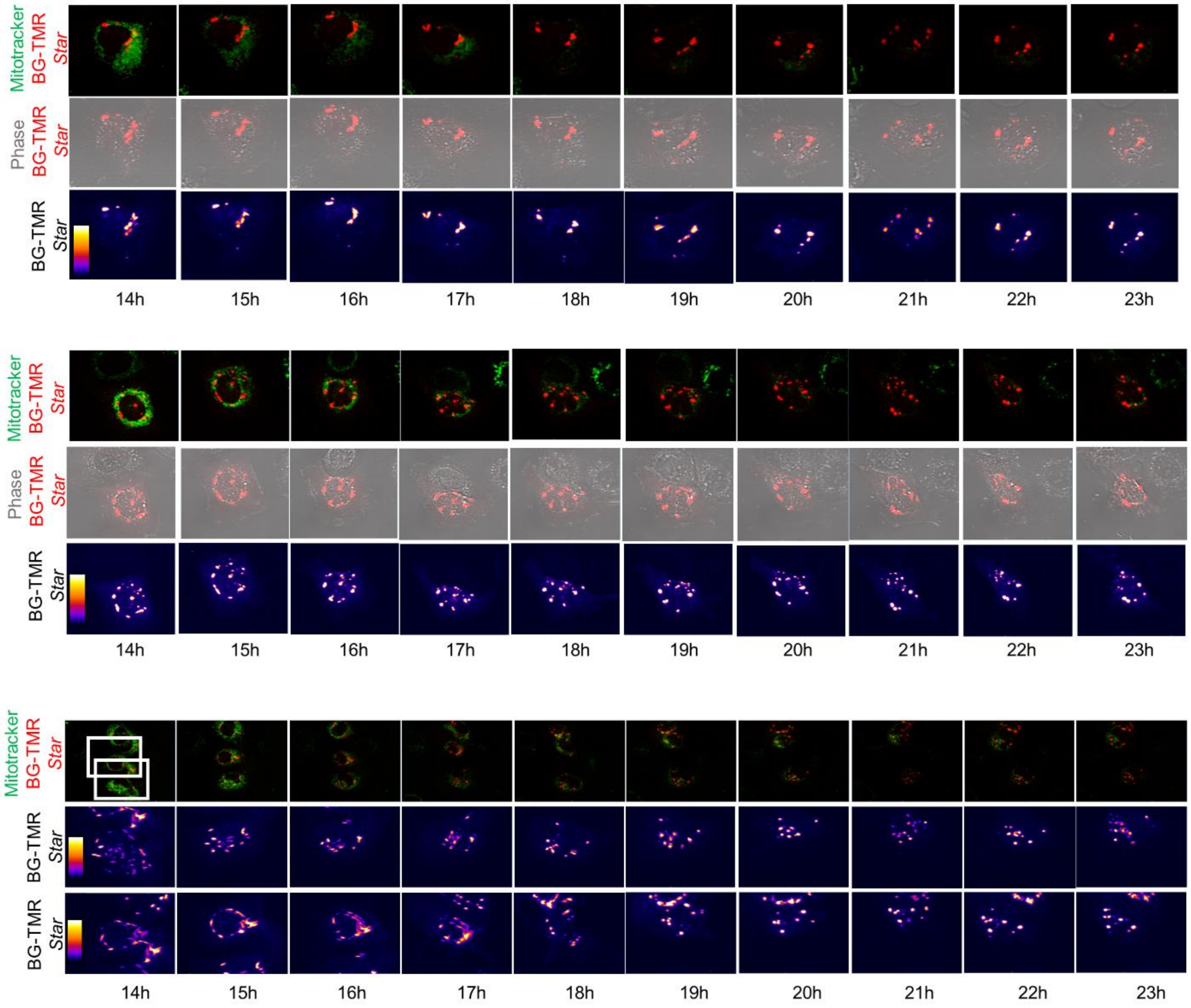

Supplementary Fig. S2 Time-lapse microscopy of cells transfected with CHIKV^{repl} $^{SNAP\text{-}P3}$, labelled with BG-TMR-*Star*. Transfected HuH-7 cells were labelled with BG-TMR-*Star* 11 hours after transfection of replicon RNA. Cells were counterstained with Mitotracker Green and imaged every hour from 14h - 23h post-transfection. Zoomed-in views of boxed regions that centre on two cells containing rod-like structures are shown.

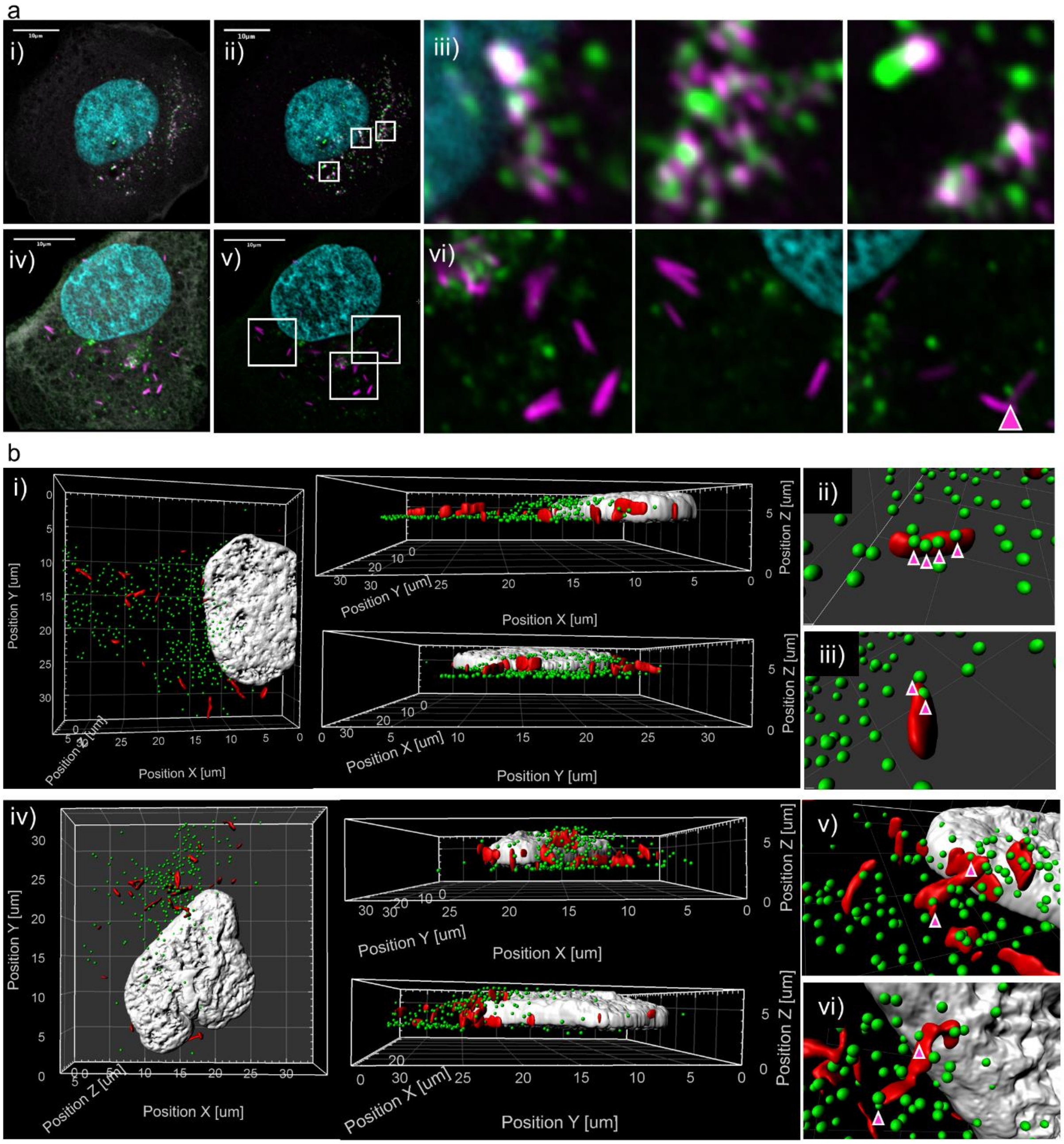

Supplementary Fig. S3 Punctate clusters of nsP3 are co-localised or associated with dsRNA foci, whereas nsP3-positive rod-like structures associate with dsRNA foci infrequently. (a) Co-localisation of SNAP-nsP3 (pseudo-coloured in magenta) and dsRNA (green) in HuH-7 cells transfected with CHIKV$^{repl\ SNAP\text{-}P3\ sg\text{-}ZsGreen}$ RNA and fixed at 12h. Images were taken in Airyscan super-resolution mode with 40 nm x 40 nm pixel

Supplementary Fig. S3 legend continued

scaling and sequential scanning of four channel tracks. Section in the top panel is towards the top of the cell, whereas the section in the bottom panel is taken from the middle of the cell. Left panels (i and iv) are overlays including ZsGreen fluorescence (grey) and cell nuclei (stained with DAPI, cyan). SNAP-nsP3 (magenta) was stained by addition of BG-TMR-*Star* to fixed cells. After methanol-permeabilisation, cells were further processed for an immunofluorescence assay with the dsRNA-specific antibody J2 (green). ZsGreen fluorescence was hidden in ii and v to emphasise the interaction between SNAP-nsP3 and dsRNA. Note an overlap between the magenta and green channels results in white colour. Numbered boxes (1-6) demarcate zoomed-in regions shown in ii and v. Magenta arrowhead points to a rod-like structure associated with a dsRNA focus. (b) Surface renderings from the 3-D reconstruction of entire Z-stacks. Two representative cells are shown (i and iv) with top and side views. Note that A and B represent cells in different fields-of-view. At 12h post-transfection dsRNA foci (green) were concentrated at the top and bottom cells replicating CHIKV$^{\text{repl SNAP-P3 sg-ZsGreen}}$. Rod-like structures with high protein density (red) were found throughout the depth of the cell. Nuclei are pseudo-coloured in grey. Right panels (ii, iii, v, and vi) are zoomed-in views of 3-D volumes in which dsRNA foci associated with nsP3-positive structures (Δ).

Supplementary Video Captions

Supplementary Video S1. 3-D visualisation of high-intensity and low-intensity nsP3 structures in a cell containing rod-like structures. Animation of the 3-D scene shown in Fig. 6a (Cell 1: Rods) was created with Imaris software. First frames of video use the volume rendering mode to display original signals from DAPI channel (coloured in cyan, 435 nm emission and 405 nm excitation) and BG-TMR-*Star* channel (coloured in grey, 585 nm emission and 555 nm excitation). Middle frames use surface rendering mode to display surfaces of high-intensity (orange) and low-intensity (pink) structures from BG-TMR-*Star* channels alongside a rendered surface of the nucleus (cyan). To illustrate differences in fluorescence intensity of nsP3 clusters, we increased the contrast sequentially in the volume rendering mode before overlaying the 3-D surfaces of low-intensity clusters (pink).

Supplementary Video S2. 3-D visualisation of high-intensity and low-intensity nsP3 structures in a cell containing granular structures. Animation of the 3-D scene shown in Fig. 6a (Cell 2: Granules). See caption of Supplementary Video S1 for additional details.

Supplementary Video S3. 3-D visualisation of high-intensity and low-intensity nsP3 structures with nsP1. Animation of the 3-D scene shown in Fig. 6b. First frames of video use the volume rendering mode to display original signals from DAPI channel (coloured in cyan, 435 nm emission and 405 nm excitation), BG-TMR-*Star* channel (coloured in pink, 585 nm emission and 555 nm excitation) and nsP1 channel (coloured

in green, 653 nm emission and 633 nm excitation). Middle frames show rendered surfaces of high-density (orange) and low-density (magenta) structures from BG-TMR-*Star* channel alongside a rendered surfaces of the nucleus (cyan) and nsP1-positive regions (green). To illustrate differences in fluorescence intensity of nsP3 clusters, we increased the contrast sequentially increased in the volume rendering mode before overlaying the 3-D surfaces of low-intensity clusters (pink). Finally, the video incorporates zoomed-in views from Fig. 6b i- v.

Supplementary Video S4. 3-D visualisation of high-density nsP3-positive structures with lipid droplets. Animation of 3-D scene shown in Fig. 7b. First frames of video use the volume rendering mode to display original signals from lipid droplet channel (coloured in green, 435 nm emission and 405 nm excitation) and BG-TMR-*Star* channel (coloured in pink, 585 nm emission and 555 nm excitation). Rendered surfaces of lipid droplets (green), high-intensity structures from BG-TMR-*Star* channel (orange), and low-intensity structures (pink) are displayed sequentially. To illustrate differences in fluorescence intensity of nsP3 clusters, we increased the contrast sequentially in the volume rendering mode before overlaying the 3-D surfaces of low-intensity clusters (pink). The video incorporates zoomed-in views from Fig. 7b, focusing on “Zoom 1” and “Zoom 2” regions.
